# Ethical issues in the COVID-19 pandemic control preparedness in a developing economy

**DOI:** 10.11604/pamj.supp.2020.35.23121

**Published:** 2020-06-29

**Authors:** Ayodele Jegede, IkeOluwapo Ajayi, Simisola Akintola, Catherine Falade, Isaac Oluwafemi Dipeolu, Simeon Cadmus, Ajala Aderemi, Abayomi Olaifa, Olufemi Olatoye, Odunayo Akinyemi

**Affiliations:** 1 Department of Sociology, Faculty of the Social Sciences, University of Ibadan, Ibadan, Nigeria; 2 Ethics Sub-committee of the Coronavirus Pandemic Response Committee, University of Ibadan, Ibadan, Nigeria; 3 Department of Epidemiology and Medical Statistics, Faculty of Public Health, College of Medicine, University of Ibadan, Ibadan, Nigeria; 4 Department of Public Law, Faculty of Law, University of Ibadan, Ibadan, Nigeria; 5 Department of Pharmacology and Therapeutics, Institute for Advanced Medical Research and Training, College of Medicine, University of Ibadan, Ibadan, Nigeria; 6 Department of Health Promotion and Education, Faculty of Public Health, College of Medicine, University of Ibadan, Ibadan, Nigeria; 7 Department of Veterinary Public Health and Preventive Medicine, Faculty of Veterinary Medicine, University of Ibadan, Ibadan, Nigeria; 8 Department of Archaeology and Anthropology, University of Ibadan, Ibadan, Nigeria; 9 Department of Veterinary Surgery and Radiology, Faculty of Veterinary Medicine, University of Ibadan, Ibadan, Nigeria

**Keywords:** Pandemic preparedness, disease control, ethics

## Abstract

Adequate preparation for highly pathogenic infectious disease pandemic can reduce the incidence, prevalence and burden of diseases like COVID-19 pandemic. An antidote to the spread of the disease is adequate preparation for its control since there is no proven curative measure yet. Effective management of identified cases, social distancing, contact tracing and provision of basic infrastructure to facilitate compliance with preventive measures, testing are proven management strategies. Although these measures seem to be the best options presently, it is important to pay attention to ethical issues arising from the implementation process to ensure best practice. While disease epidemic is not alien to human societies, lessons from previous outbreaks are vital for addressing future outbreaks. For effective control of this pandemic, there should be a clear definition of social distancing in terms of distance and space in line with the WHO definition, adequate provision of basic amenities, screening and testing with specific criteria for selecting those to be screened. Also, there should be a free testing procedure, access to treatment opportunities for those who test positive, ethical free contact tracing practice, respect for the autonomy of those to be tested, and global best practice of open science, open data and data sharing practices. In conclusion, a framework/guideline for epidemic/pandemic ethics guidance should be developed while an ethical sensitive communication manual should be prepared for public engagement on epidemic and pandemic.

## Commentary

**The thesis:** adequate preparation for highly pathogenic infectious disease pandemic can reduce the incidence, prevalence and burden of diseases like COVID-19 pandemic [1]. Coronavirus disease, also referred to as COVID-19, is an emerging respiratory disease in the human population and a new variant of Coronavirus SARS-CoV-2 [2]. The virus, first detected in December 2019 in the China city of Wuhan, is highly contagious [2]. Its main clinical symptoms include fever, dry cough, fatigue, myalgia and dyspnea. In China, 18.5% of the patients with COVID-19 progressed to a severe stage [2], characterised by acute respiratory distress syndrome, septic shock, difficult-to-tackle metabolic acidosis, bleeding and coagulation dysfunction [3]. An antidote to the spread of the disease is adequate preparation for its control since there is no proven curative measure yet. Other preventive measures include social distancing, contact tracing, availability of basic infrastructure to facilitate compliance with preventive measures, testing and data sharing [3]. Implementation of these measures needs some ethical considerations.

**Context:** the novel human coronavirus infection is associated with the respiratory system, specifically the epithelial cells of the lung with an incubation period of 1-14 days. The symptoms manifest after about 5.2 days in some patients [2]. Direct contact with an infected person and droplets of respiratory pathogens or urine and faeces are believed to be the primary modes of transmission. Age, sex and underlying health conditions are the risk factors for complications or premature death [2]. The death of healthy young persons with no known pre-existing health conditions in the United Kingdom and the United States of America raises a serious concern about the disease mechanism. The daily increase in death toll and new cases of COVID-19 with its concomitant effects on the socio-economic and political landscapes are major concerns globally. To prevent new cases, social distancing and self-isolation are major considerations. This has made many countries around the world to close borders, while some declare complete lockdown [4]. In line with global best practices, the Nigerian government at Federal and State levels have equally followed suit, resulting in a devastating socio-economic backlash on communities that are mostly self-employed, due to lack of existing evidence-based lockdown policy. This scenario is the same in many African and low socio-economic countries. While countries might have leveraged the lessons from the previous Lassa fever and Ebola epidemic in addressing the current situation [5], the novelty of COVID-19 is challenging the pre-existing experiences.

**Lessons from previous epidemics:** while disease epidemic is not alien to humanity, lessons from previous outbreaks are vital for planning during similar occurrences. The public awareness programme was vital in the containment of the Ebola (EVD) outbreak in Nigeria. The heroic act of professionalism displayed by Nigerian physicians and other health workers, especially with the index case, was very instrumental in containing the spread of the disease. Similarly, institutional responses helped to control the epidemic and reduce citizens´ exposure. Also, the government´s response to Ebola outbreak built public confidence in the actions rolled out to contain the spread of the disease. However, one thing is to learn lessons from the previous experience, and it is another to utilise the lessons learned. The initial transmission of Ebola in Nigeria was through contact with a foreigner (the first index case) who introduced the disease into the country. Similarly, COVID-19 was transmitted into Nigeria through the same process. Second, Nigeria did not upgrade its methods and lessons from the contact-tracing experience of Ebola whereby some persons who might have been exposed to the virus may not have been unaccounted for. Third, the infection of health workers which was experienced during the Ebola epidemic has also been reported in the current pandemic [6]. Fourth, little or no lesson is learnt about the effective management of the isolation centres leading to some quarantined persons´ unwillingness to stay in those centres. Fifth, proper hygiene is central to the control of this pandemic, but this cannot be achieved in the absence of basic infrastructures like potable water and electricity supply. This is still a significant challenge in poor resource settings. This has implication for handwashing and regular drinking of water. Irregular electricity supply in Nigeria poses a challenge to disseminating appropriate public health messages, leaving public health practitioners to rely on the limited coverage of handbills, unlike the more effective electronic medium of mobile telephone, TV, radio, that has broader acceptance and coverage. Lastly, data sharing is still a major problem among the agencies involved in control activities. This is due to poor data sharing management, inadequate regulatory framework and health information exchange policy. The import of this is that if lessons learned were adequately utilised, a number of the challenges faced by most African and countries today would have been averted.

**Ethical challenges:** medical ethics is about the values that should be respected by all healthcare workers while interacting with individuals, families and communities. Ethics may sometimes be considered a scary term by some healthcare professionals because it is a word that may bring to mind an accusation of wrongdoings or mistakes, but this is not the case [7].

Ethical theories: ethical decision making is an action-based expression of day-to-day living. In every society, human interaction is guided and sustained by the actions of the actors, which is adjudged ethical or not. It is considered ethical when an action is right and vice versa. Every action is based on the intention of an actor that normally results in some consequences. For a critical evaluation of a phenomenon, ethics can focus on the action, the actor, the intention or the consequences. As individuals, we have a duty to one another. This duty is based on the societal norm. Norm is central to the duty ethics referred to as deontology. An action is defined morally right by the moral norms or rules of society. During epidemic control, every frontline staff has a duty to the population being served. Every actor must recognise his or her duty and obligations to other fellow human beings and those expected from others in the course of discharging his official responsibilities. Immanuel Kant´s duty ethics, known as the Kantian theory, is interested in autonomy and argues that an actor should place a moral norm upon himself and obey it. In carrying out a duty, actors should not lose sight of the consequence of such an action. This is why ethics also pay attention to the intention of an action. This directs attention to the outcome of every action. One type of consequentialism is utilitarianism, founded by Jeremy Bentham. In utilitarianism, the consequences of actions are measured against one value such as “happiness, welfare or pleasure” resulting from an action. Utilitarianism is based on the utility principle. That is simply of need to give the greatest happiness to the highest number of people. An action is morally right if it results in pleasure, whereas it is wrong if it gives rise to pain. In the context of epidemic control, the consequence of control measure should not lead to pain or hurt but, the one that will benefit the generality of the population. Aching, this depends on how an individual actor exhibits the right virtue. An actor should base his or her actions on the right virtues of “selflessness, caring, love and charity”. So, virtue ethics suggests that frontline staff should take on the morally good and responsible character. Virtue ethics is rather similar to duty ethics. Duty ethics is based on certain rules/norms, while virtue ethics is based on certain virtues. As indicated by Aristotle, every moral virtue is positioned somewhere between two extremes in which the correct moral virtue equals the optimal balance between these two extremes. Appropriate behaviour will be guided by care exhibited in the course of carrying out a duty. Care ethics emphasises that the development of morals is not caused by learning moral principles but that norms and values should be learned in specific contexts like during epidemics. Ethics of managing epidemic control is different from clinical practices and public health practices. Empathy is emphasised in care ethics by contacting other people, and by placing oneself in other people’s shoes to understand what they are experiencing at a particular point in time. Every action must be built on maintaining the relationships between people. Applying these theories to this discussion will be enhanced by ethical principles.

**Ethical principles:** in measuring an action, ethical principles have key indicators in this direction. According to Beauchamp and Childress [8], there are four basic principles of healthcare ethics namely autonomy that is, the right for an individual to make his or her own choice; beneficence that is, the principle of acting with the best interest of the other in mind. Others are non-maleficence that is, the principle that “above all, do no harm,” as stated in the Hippocratic Oath; and justice. Actor´s duty must be exhibited within the ambit of these ethical principles for it to be adjudged ethical. These should be considered while rolling out interventions to the people. Even though individual choices are legally limited during epidemics and pandemics, it is also important to ensure that human dignity is not eroded. It must be done with justice and fairness; every member of society should be treated equally. Risk should be minimised in the process of implementing pandemic control activities. Public trust is important and should not be eroded during epidemic and pandemic control, and this is made possible through truth-telling and promise-keeping. All these are important considerations for ethical free pandemic control implementation. The COVID-19 pandemic is worrisome because of the high morbidity and mortality rates in addition to the accompanying socio-economic and psychological burden. While the treatment and control of the disease decisions fall within the purview of policymakers and healthcare workers, the legal, cultural, socio-economic and psychological effects are issues that bother on the people [9]. Many of these issues have manifested in the last few weeks of implementing preventive measures for the pandemic, ranging from complaints about social distance, isolation, lockdown, testing, lack of basic amenities, especially water and electricity, contact tracing and data sharing. Assessing the ethical basis of these issues becomes necessary in the face of human rights and social justice.

**Social distancing:** while social distancing is a proven solution for limiting the spread of COVID-19, it may be challenging due to the context of where it is being implemented. Although the distance measurement of two metres - 2 yards and 6 feet - is clearly stated in many public health education documents, adequate understanding may be a challenge in many low and low-middle income countries where literacy level is still very low. Identifying space may be a major challenge in highly overcrowded communities and quest to continue communal living. While the nature of space, whether enclosed or open, is not explicit, the exemptions are also not clearly defined. It is expected that a safe distance of spacing should be put in place by all when interacting at every location. In real-time, people, especially in commercial centres and markets, have chosen to exempt themselves from this preventive measure based on their perception. This is why there has been arguments and counter-arguments about the strategies for easing the lockdown. Some do not see the reason why markets and business should open while religious and schools should not open. Where is the place of the individual to make a choice in this context when everything is predetermined? To what extent is individual acting with the best interest of others in mind in a situation where many cannot determine the appropriate safe distance? Is it fair to apply a common sanction to everyone for failing to obey social distancing when some lack adequate education to make good sense of what equates to 2 meters? All these are ethical dilemma arising from the pandemic.

**Isolation:** while isolation is not alien to Africa for disease control, it is worrisome that the current practice of isolation has raised many uproars. The question here is to what extent does an individual have a say in a decision affecting his or her life? Do people have the choice of where and how to be isolated? To what extent is the managers of isolation centres acting with the interest of other patients in mind when everyone who tested positive but with varying degree of life circumstances is accommodated in the same place? How much support do patients receive during the isolation period? In the traditional setting, the individual is supported through communal way of living, which makes survival, economically and psychologically, bearable during periods of isolation. Ethics of restricting sociation or freedom of interaction is an important consideration in this context. The contemporary situation is a product of erosion of the traditional value. The complete breakdown of the organic social relationship of traditional communal living in a country like Nigeria has contributed to its already high ranking in the poverty index [10]. The good of society is paramount at such a period like this, and the state has no option but to restrict the rights of the people. The society should first be educated on this, and the law governing the rule should have been spelt out to the people more clearly in the language they understand at the time the social distancing policy was announced to prevent the revolt and non-compliance. This communication gap brought about a delay in compliance with social distancing, which was also observed at the top level of the government and may “fired” up the transmission of COVID-19 in Nigeria.

**Contact tracing:** contact tracing is critical to effective control of any pandemic. This has been a challenge with the current pandemic due to lack of preparedness and adequate information leading to inability to trace some people who have been exposed one way or the other as they might have provided wrong contact details on arrival in the country. This is due to fear of stigmatisation and forceful isolation in special isolation centres which are considered unfit for human habitation. Many contacts may not accept that they are a risk to others, especially if they are asymptomatic. Arguments of balancing individual autonomy and the good of the society come into play.

**Testing:** related to contact tracing is testing for the virus infection. Aside from the stigma that comes with testing positive to COVID-19, individuals need to have clear information about the procedure for testing ahead of the test. In the last few months of the control efforts, lack of preparedness for testing was reported at some designated centres. The question of equity in testing is important. People being tested are more of the elites which make people view the disease as an elite disease. Even when a patient tests positive, what about their primary and secondary contacts? How do we account for the contacts in society with no data about citizens? What incentive is available for people to volunteer information about their contact with a known patient? While some individual may want to volunteer information about themselves, this may be hindered by a lack of proven effective treatment. While the elites may volunteer, the poor may not. With the current strategy of testing, it may be challenging to trace contact effectively, which may increase the burden of community transmission.

**Data sharing:** lack of data sharing may hinder effective control of epidemics. The conflicting figure is a sign of a lack of adequate preparation for the pandemic. It also portrays a lack of coordination which could bring about a breakdown in responses to epidemics and pandemics. Also, there are insinuations that the figures presented are likely to be inaccurate, and this gives people a false impression about the gravity of the epidemic which may militate against compliance with the preventive measures and risk perception. The perceived under-reporting can also be partially explained by the very low number of tests being done, especially as a significant proportion of infected persons do not have overt symptoms that could make them stay at home or seek medical attention. To support mitigating or addressing the gaps identified in the ongoing efforts of the government to contain the COVID-19 pandemic, it is pertinent to proffer some solutions and advice.

**Solutions:** for effective control of this pandemic, the government will benefit from addressing the following issues: first, efforts should be intensified to encourage people to adhere to social distancing directive. The enforcement agencies like the police force should respect people´s human rights while carrying out their duties and should educate on the need to comply where they default. Force or cohesion should be the last resort. The government needs to put in place facilities for self-isolation to accommodate people in low/lower-middle-class. Especially where whole families live in one or two bedrooms with next-door apartments, only separated by a wall, where a single building could accommodate more than one family. Public awareness and education should be very clear about social distancing to enhance compliance. Second, adequate provision of basic amenities, like potable water and electricity for proper maintenance of hygiene at the individual, community and institutional levels, is important. The provision of food and sanitisers by the government is laudable; however, the reach is still abysmal, and the promise should be kept. Communal efforts at providing some of these essential needs may be complementary. Third, screening and testing are essential, but specific criteria for deciding on selecting those who should be screened should be logical and be made public. Those who are not qualified should be provided with adequate communication and assured support. Fourth, those who test positive of the virus should be given access to treatment opportunities in a conducive environment, learning from the HIV and Ebola epidemics experiences. Fifth, testing should remain free, and the government should make it available to all, regardless of setting or status. Community testing is now being carried out in some African countries. Nigeria should be able to do the same as the benefits of such a policy are numerous. Apart from citizens knowing their COVID-19 status, it will provide excellent surveillance data.

Sixth, on the ethical issues surrounding contact tracing, there should be no infringement of privacy and rights of the contact to be traced. The privacy, dignity, cultural and customary rights should be respected. Information collected from individuals and community should be kept strictly confidential to avoid COVID-19-related stigmatisation. It is not uncommon that neighbours may suspect or perceive every unsolicited visit to suspected individuals by health officials saddled with the responsibility for contact tracing and may bring about community rumour. This should be addressed by providing adequate education on contact tracing to the general populace. Information about the extant law governing the need to breach confidentiality for societal good should any contact or person who tests positive refuse to cooperate should be clearly explained to the general public. Seventh, who decides whether or not to be tested for the virus? How is the autonomy of the individual affected in this context? Is it equitable? These should be in line with existing international and national guidelines and ensure compliance with best practice. Eighth, the current global practice is open science, open data and data sharing which has become the global standard. Healthcare workers involved in contact-tracing must be cautious of being infected in the process. There should be a clear guideline on how to harness data about this category of people in the larger database without breaching the ethics of confidentiality. On COVID-19 data collection, ownership of data and at what point the data be shared should be addressed. There should be some exemptions on harnessing data from any contact traced for the societal good. Statistical/mathematical modelling for projecting the progress of the pandemic should be done truthfully, and the information arising therein shared with the public should reflect the true situation. Dissemination should be done at different levels using communication modalities appropriate for each level. Based on lessons from previous epidemics, the government should take into consideration the cultural context to amend the public order in a way that will be culturally sensitive without compromising the global best practices. In doing this, the government may leverage the indigenous disease prevention or seclusion practices (such as done in the time of smallpox and leprosy) to drive home the points for the current pandemic control measures. Also, there are specific characteristics of each epidemic and its causative pathogen that must be carefully studied so that transmission peculiarities can be used to modify and improve containment measures.

**The of ethics committee:** the COVID-19 raises new concerns for research. Unlike before the pandemic, social distancing has to redefine research context. While researchers and research participants may not know each other due to social distancing, the myth of one-to-one interaction and knowledge gained from non-verbal cues will be missing in data analysis. When an interview is conducted on phone or online, the researcher does not know whether or not people assisted the respondent to answer the question(s). As a result, research design needs to take into consideration the pandemic and reflect all necessary conditions related to it in the study. While researchers must obtain ethics approval before conducting any biomedical research, including COVID-19 related research, the ethics committee should ensure that research protocols address the current realities. To achieve this, ethics committee members should be trained on how to review research protocols in the context of the pandemic.

## Conclusion

Preparedness is critical in pandemic control. In the case of COVID-19 pandemic, there seems to be inadequate preparedness in poor resource settings like Nigeria. The situation is complicated by not putting lessons from the previous epidemics into consideration when planning control and prevention activities for a new epidemic/pandemic. It is high time that the government took infectious diseases control and prevention seriously and built up the country´s health systems infrastructure and human capacities in preparation for diseases of public health emergencies. This is necessary because of the seemingly lack of adequate modern medical technologies and personnel to address the situation in the face of a full-blown outbreak. Disease control managers should know that control measures may be resisted if they are contextually insensitive. One of the critical outcomes of this pandemic is that a framework/guideline for epidemic/pandemic ethics guidance should be developed. Also, an ethical sensitive communication manual should be developed for public engagement on epidemic and pandemic.

## Competing interests

The authors declare no competing interests.

## Authors’ contributions

Ayodele Jegede conceptualised the idea of the paper and critically discussed by IkeOluwapo Ajayi, Simisola Akintola, Isaac Oluwafemi Dipeolu, Simeon Cadmus, Aderemi Ajala, Abayomi Olaifa and Olufemi Olatoye for adoption as a group project. Ayodele Jegede, IkeOluwapo Ajayi and Simisola Akintola conducted the investigation that resulted into this publication while Ayodele Jegede curated and analysed the data. Ayodele Jegede, IkeOluwapo Ajayi and Simisola Akintola worked on the methodology while Ayodele Jegede was the project administrator and supervised the process that led to the writing of this paper. Ayodele Jegede, IkeOluwapo Ajayi and Simisola Akintola provided the resources for the publication while Ayodele Jegede wrote the original draft of the article. Ayodele Jegede, IkeOluwapo Ajayi, Simisola Akintola, Catherine Falade, Isaac Oluwafemi Dipeolu, Simeon Cadmus and Odunayo Akinyemi reviewed and edited the drafts. All the authors have read and agreed to the final manuscript.
